# Field survey of support groups for people with neurodevelopmental disorders in Japan

**DOI:** 10.1002/pcn5.70028

**Published:** 2024-11-04

**Authors:** Sayo Hamatani, Yoshifumi Mizuno

**Affiliations:** ^1^ Research Center for Child Mental Development University of Fukui Fukui Japan; ^2^ Division of Developmental Higher Brain Functions, United Graduate School of Child Development University of Fukui Fukui Japan; ^3^ Department of Child and Adolescent Psychological Medicine University of Fukui Hospital Fukui Japan

## Abstract

This study conducted a systematic review of support groups currently active in accommodating individuals with neurodevelopmental disorders in Japan. The characteristics of the 86 approved support groups for neurodevelopmental disorders are detailed in the Supplementary Materials.

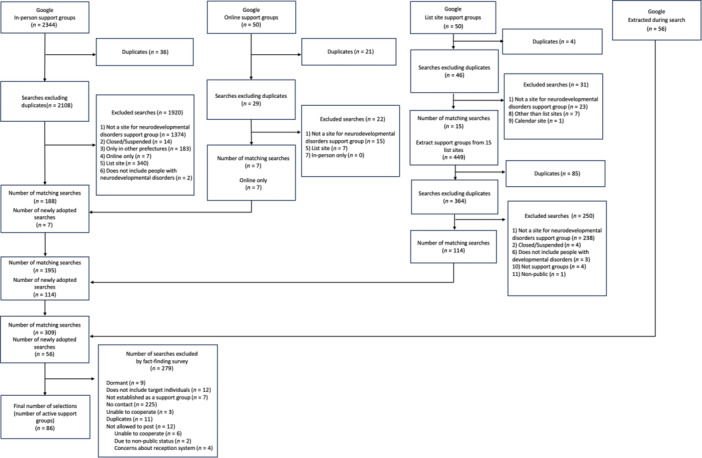

People with neurodevelopmental disorders (NDDs) face many difficulties in their daily and social lives, and without appropriate support, their risk of developing secondary disorders increases.^1^, ^2^ These secondary disorders can range from depression and anxiety disorders to social isolation, which are associated with additional complications.^3^, ^4^ Therefore, it is necessary to establish a system through which people with NDDs can receive appropriate support. Support groups that serve people with NDDs play an important role in the community and provide space for people to gather, share experiences and information, and support one another.^5^ Moreover, it has been reported that reducing social isolation and providing education on life skills may improve the quality of life among people with NDDs.^6^, ^7^, ^8^ However, to date, no studies have systematically evaluated these support groups for people with NDDs in Japan. Hence, this study aimed to systematically survey and list support groups for individuals with NDDs.

A systematic search was conducted using Google's search engine to identify support groups for people with NDDs. In this study, based on the Act on Support for Persons with Developmental Disabilities, we focused on support groups that assist people with autism spectrum disorder, attention‐deficit/hyperactivity disorder, and learning disorder. The searches were conducted from November 1, 2023, to January 22, 2024. This search was conducted in multiple rounds to ensure a comprehensive review of available support groups. This study did not set specific age restrictions and included people ranging from children to adults. During each search, if the number of results exceeded 50, the top 50 results were selected for review. For the Japanese search terms used, refer to File S1. The first search term entered was “neurodevelopmental disorders, support group, the name of 47 prefectures.” Then, duplicate returns were excluded, and the following exclusion criteria were applied: (1) not a site for an NDDs support group, (2) closed/suspended support groups, (3) support groups outside of the defined prefectures, (4) online support groups, (5) composite lists of support group sites, and (6) no inclusion of people with NDDs. The second search term entered was “neurodevelopmental disorders, support groups, online,” to capture support groups available online. Exclusion criteria included (1) and (5) above, and (7) support groups providing face‐to‐face support. Next, for the sites listing support groups, we used “neurodevelopmental disorders, support groups, list,” and excluded any results corresponding to (1) above, as well as (8) support groups other than the list sites, and (9) calendar sites. Subsequently, support groups were extracted from the extracted list sites based on the exclusion criteria:(1), (2), (6), (10) not a support group, and (11) closed to the public. In addition, support groups newly found in the search process were also extracted. Finally, among the 367 support groups extracted, active support groups were identified by excluding those that could not be contacted after two attempts via phone, e‐mail, or social networking site, among other reasons (Figure 1).

**Figure 1:**
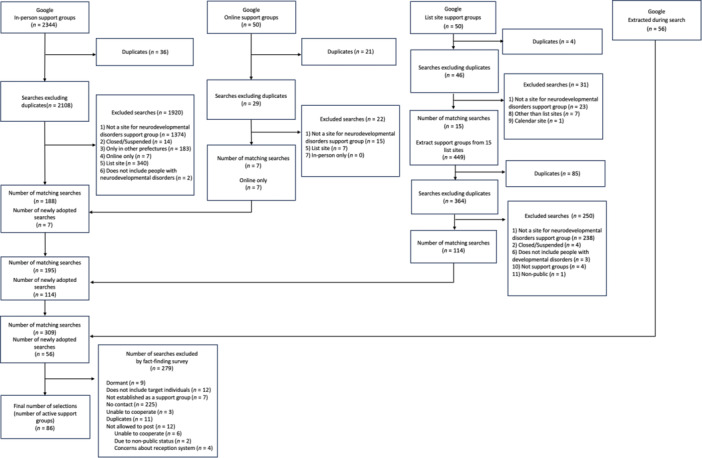
Flow diagram of study selection and exclusion criteria. This figure presents the flow diagram of the systematic search process, detailing the inclusion and exclusion of sites from initial identification to final selection. A detailed description of some of the exclusion criteria:(1) “Not a site for NDDs”: Excludes sites primarily serving as advertising platforms, newspaper websites, or blogs by individuals who do not run support groups, as well as sites of groups that do not focus on neurodevelopmental disorders (NDDs). (3) “Only in other prefectures”: Excludes sites of support groups not located within the specified prefecture under study. For example, when searching for support groups in Hokkaido, any sites from other prefectures except Hokkaido are excluded. This filtering is consistently applied across all 47 prefectures in Japan. (5) “List site”: Excludes websites that compile lists of support groups across multiple prefectures to ensure that data specific to each targeted prefecture are extracted. Unique groups listed only on these aggregate sites are, however, extracted using the search term “NDDs support group list.” (9) “Calendar site”: Excludes sites that manage support group schedules in a calendar format due to their high content variability, which changes with each access, complicating stable data extraction.

Following the review, 86 active support groups were included in this analysis. Characteristics of the included support groups are available in File S1 as a list of support groups for NDDs. The number of active support groups per prefecture ranged from 0 to 14, with 12 prefectures (26%) having no support groups. While the number of support groups in urban areas, such as Tokyo and Osaka, accounted for approximately one‐quarter of the total, the number in rural areas was small, indicating a large regional maldistribution of support groups. The most frequent meeting frequency was once a month (43%), followed by once every few months (17%) (Supporting Information S1: Figure S1). While 64% of the meetings were held in person, only 6% were held exclusively online, and 44% were held both in person and online (Supporting Information S1: Figure S2). Free exchange was the main activity in more than 95% of the support groups, followed by learning and information gathering in 53 (62%) and lectures in 37 (43%) (Supporting Information S1: Figure S3). In contrast, only 15 (17%), or approximately one‐sixth of the total number of support groups, conducted social skills training.

To our knowledge, this study is the first attempt to systematically survey active support groups for people with NDDs in Japan. The resulting list of support groups should prove to be a useful resource for people with NDDs looking to receive appropriate support. Providing detailed information on the activities and the delivery method of these groups is expected to ease the process of identifying support that meets the needs of individuals with NDDs. However, further investigation is needed, as the quality and effectiveness of the selected support groups are unknown.

## AUTHOR CONTRIBUTIONS

**Sayo Hamatani:** Writing—original draft; conceptualization; methodology; searching and extracting the data; data curation; analysis; project administration; funding acquisition. **Yoshifumi Mizuno:** Project administration; supervision; writing—review and editing; funding acquisition. All authors read and approved the final manuscript.

## CONFLICT OF INTEREST STATEMENT

The authors declare no conflict of interest.

## ETHICS APPROVAL STATEMENT

This research did not require an ethical review as it did not fall under the ethical guidelines for life science and medical research involving human subjects.

## PATIENT CONSENT STATEMENT

All the support groups provided informed consent to participate in the study.

## CLINICAL TRIAL REGISTRATION

N/A.

## Supporting information

Supporting information.

Supporting information.

## Data Availability

A list of support groups for NDDs is freely available in the Supplementary Material.
