# Supplementary material for: Field survey of support groups for people with neurodevelopmental disorders in Japan
Source: PCN Rep. 2024 Nov 4;3(4):e70028. doi: 10.1002/pcn5.70028 (PMC11532993; doi:10.1002/pcn5.70028)
Supplement: Supplementary file 1 — Supporting information. [file PCN5-3-e70028-s002.docx]

**Supplementary Material**

Japanese Search Terms for Neurodevelopmental Disorders Support Groups：

"発達障害 当事者会 北海道", "発達障害 当事者会 青森県", "発達障害 当事者会 岩手県", "発達障害 当事者会 宮城県", "発達障害 当事者会 秋田県", "発達障害 当事者会 山形県", "発達障害 当事者会 福島県", "発達障害 当事者会 茨城県", "発達障害 当事者会 栃木県", "発達障害 当事者会 群馬県", "発達障害 当事者会 埼玉県", "発達障害 当事者会 千葉県", "発達障害 当事者会 東京都", "発達障害 当事者会 神奈川県", "発達障害 当事者会 新潟県", "発達障害 当事者会 富山県", "発達障害 当事者会 石川県", "発達障害 当事者会 福井県", "発達障害 当事者会 山梨県", "発達障害 当事者会 長野県", "発達障害 当事者会 岐阜県", "発達障害 当事者会 静岡県", "発達障害 当事者会 愛知県", "発達障害 当事者会 三重県", "発達障害 当事者会 滋賀県", "発達障害 当事者会 京都府", "発達障害 当事者会 大阪府", "発達障害 当事者会 兵庫県", "発達障害 当事者会 奈良県", "発達障害 当事者会 和歌山県", "発達障害 当事者会 鳥取県", "発達障害 当事者会 島根県", "発達障害 当事者会 岡山県", "発達障害 当事者会 広島県", "発達障害 当事者会 山口県", "発達障害 当事者会 徳島県", "発達障害 当事者会 香川県", "発達障害 当事者会 愛媛県", "発達障害 当事者会 高知県", "発達障害 当事者会 福岡県", "発達障害 当事者会 佐賀県", "発達障害 当事者会 長崎県", "発達障害 当事者会 熊本県", "発達障害 当事者会 大分県", "発達障害 当事者会 宮崎県", "発達障害 当事者会 鹿児島県", "発達障害 当事者会 沖縄県", "発達障害 当事者会 オンライン", "発達障害 当事者会 一覧"

Figure S1. Frequency of meetings

Figure S2: Number by meeting methods

Figure S3: Number by activities
