# Supplementary material for: Field survey of support groups for people with neurodevelopmental disorders in Japan
Source: PCN Rep. 2024 Nov 4;3(4):e70028. doi: 10.1002/pcn5.70028 (PMC11532993; doi:10.1002/pcn5.70028)
Supplement: Supplementary file 2 — Supporting information. [file PCN5-3-e70028-s001.pdf]

発達障害当事者支援団体リスト

2024年版　ver.1.0

発達障害当事者支援団体リスト

リストについて

社会的スキルを身につけ、生活の質（QOL）を高めていくためには、周りからのサポートが非常に重要です。私たちは「発達障害を持つ当事者を受け入れている、現在活動中の団体」を体系的にレビューし、掲載許可が得られた86の支援団体をリストアップしました。このリストには主に当事者会が含まれていますが、家族会なども掲載されています。1人で悩んでいる方や、当事者へ支援団体を紹介したい方は、このリストをぜひご利用ください。なお、支援団体の情報は、2024年5月時点の情報であり、最新の状況とは異なる可能性があります。支援団体のHPやSNS等で必ずご確認ください。

本研究は、日本学術振興会（JSPS）の科研費（課題番号 22H00985）および公益財団法人武田科学財団からの助成を受けて、福井大学の濱谷沙世特命助教（研究執筆担当者）、水野賀史准教授、小林未歩さん、北川慶子さん、黒田美優さん、野上佳南さん、石川瑞菜さんの協力を得て行われました。

免責事項

1. 全ての支援団体が載っているわけではありません。研究のある一定の基準に合わせて、体系的に調査しました。その基準に該当しなかった支援団体は抽出されていません。また、掲載許可が降りていない支援団体は載っていませんのでご了承ください。
2. 支援団体の相談について受け付けておりませんので、ご了承ください。
3. リンク先のホームページや支援先は当サイトが管理運営するものではありません。
   支援団体の信頼性などについて当サイトは責任を負いません。
4. 症状の悪化やトラブルなど、本サイトやリストの利用により生じたあらゆる損害に対する責任は一切負わないものとします。あらかじめ、ご了承ください。

北海道 ｜ 発達障害の当事者ミーティング こんとん

当事者会　専門家のサポート体制あり

開催場所	札幌市社会福祉センター、函館青年センターなど
連絡先	070-5612-7910<br>fuku@conton.net
支援先のHP	https://www.conton.net/
参加条件：当事者会	発達障害特性の当事者<br>特記すべき条件なし（付き添い）
参加条件：家族会	開催なし
参加条件：診断の有無	なし
開催	月に2回／対面
参加費	有料（札幌　初回1000円、二度目から400円）<br>（函館　夏300円、冬400円）
参加方法	直接訪問可（予約連絡不要）<br>＊初回の連絡も不要
SNS	なし

活動内容

○ 自由交流	講演会	集団療法
○ 学習・情報収集	SST	レクリエーション
心理教育	ペアトレ	その他

備 考

北海道 ｜ 北海道凸凹の会

当事者会

開催場所	札幌市内の区民センターもしくはレンタルスペース
連絡先	dekoboko2017-hokkaido@yahoo.co.jp
支援先のHP	https://ameblo.jp/dekoboko2017-hokkaido/
参加条件：当事者会	発達障害特性、理解のある当事者<br>特記すべき条件なし（付き添い）
参加条件：家族会	開催なし
参加条件：診断の有無	なし
開催	月に1回／対面
参加費	有料500円（内容によっては変動あり）
参加方法	事前連絡必須（予約・連絡必須）
SNS	X @dekoboko2017ms

活動内容

○ 自由交流	講演会	集団療法
○ 学習・情報収集	SST	○ レクリエーション
心理教育	ペアトレ	○ その他（ボードゲーム、カードゲーム）

備 考　未成年者の参加は要相談

北海道 ｜ 札幌PDD広汎性発達障害ピアサポート協会

当事者会　専門家のサポート体制あり

開催場所	北海道小樽市、札幌市エリア
連絡先	maverickjapan2020@gmail.com
支援先のHP	https://peraichi.com/landing_pages/view/sppsa
参加条件：当事者会	発達障害特性の当事者
参加条件：家族会	開催なし
参加条件：診断の有無	あり
開催	不定期（開催しない年もある）／対面
参加費	PDD当事者会員:入会金1000円<br>年会費3000円
参加方法	事前連絡必須（予約・連絡必須）
SNS	なし

活動内容

○ 自由交流	講演会	集団療法
学習・情報収集	SST	レクリエーション
心理教育	ペアトレ	○ その他（散歩や写真撮影など）

備考

北海道 ｜ 北海道自閉症協会道南分会

家族会　専門家のサポート体制あり

開催場所	函館市中央図書館、函館市亀田交流プラザ
連絡先	090-9430-1348（10：00～16：00）<br>info@asj-dounan.org
支援先のHP	https://asj-dounan.org/
参加条件：当事者会	開催なし
参加条件：家族会	特記すべき条件なし<br>＊ただし当事者は会員制
参加条件：診断の有無	あり
開催	①おしゃべり広場　2～3ヶ月に1回／対面<br>②公開講座　年に2～3回 ／対面<br>③自閉症サロン（会員対象）年に8回／対面
参加費	有料：正会員【入会金】3000円<br>【年会費】7000円（一人親世帯は3500円）<br>賛助会員【入会金】無料 【年会費】1口2000円～
参加方法	事前連絡必須（予約・連絡必須）
SNS	なし

活動内容

○ 自由交流	○ 講演会	集団療法
○ 学習・情報収集	SST	レクリエーション
心理教育	○ ペアトレ	○ その他（※備考参照）

備考

全国に同団体あり
会員の条件（正会員：北海道在住で、自閉症の診断を受けた子どもの保護者 / 賛助会員：活動に同意された方）
※ 啓発活動、就労・生活場所の見学・学習会、世界自閉症啓発デーin Hakodate

北海道 ｜ 北海道自閉症協会十勝分会

家族会

開催場所	〒080-2474 北海道帯広市西24条南1丁目32-6		
連絡先	waiwaiclub@bb.wakwak.com	支援先のHP	なし
参加条件 当事者会	開催なし	開催	不定期（開催しない年もある）／対面、オンライン
参加条件 家族会	特記すべき条件なし<br>＊ただし、当事者は発達障害特性、地域限定	参加費	有料　年会費4500円<br>入会金3000円
参加条件 診断の有無	なし	参加方法	事前連絡必須（予約・連絡必須）
SNS	なし		

活動内容

○ 自由交流	○ 講演会	集団療法
○ 学習・情報収集	SST	レクリエーション
心理教育	ペアトレ	その他

備考：全国に同団体あり

青森県 ｜ 青森県発達障がい者支援センター「ステップ」

発達障がい者支援センター　専門家のサポート体制あり

開催場所	発達障がい者支援センター事業所の施設内など<br>〒030-0822　青森県青森市中央３丁目20-30県民福祉プラザ3階		
連絡先	017-777-8201<br>aoshien6@adagio.ocn.ne.jp	支援先のHP	なし
参加条件 当事者会	開催なし	開催	※備考参照／対面、オンライン
参加条件 家族会	発達障がい、その疑いのある児者のご家族	参加費	無料
参加条件 診断の有無	なし	参加方法	事前連絡必須（予約・連絡必須）
SNS	なし		

活動内容

○ 自由交流	○ 講演会	集団療法
○ 学習・情報収集	SST	レクリエーション
心理教育	○ ペアプロ・ペアトレ	その他

備考：
全国に同団体あり
※開催（不定期であり、開催しない年もある）
①家族対象茶話会　年に2回　/②ペアレントメンターによる傾聴事業 年に６回
③ペアレント・プログラム事業 年に１～２回　/　④ペアレント・トレーニング事業 年に１～２回

岩手県 ｜ ふらっと寄れる小さな居場所「cocomo」、オンラインおしゃべり会「縁が輪」 特定非営利活動法人ココカラいわて

当事者会　専門家のサポート体制あり

開催場所	岩手県盛岡市内		
連絡先	kokokaraiwate@gmail.com	支援先のHP	https://www.kokokaraiwate.com/
参加条件：当事者会	特記すべき条件なし	開催	月に2回／対面、オンライン
参加条件：家族会	開催なし	参加費	無料
参加条件：診断の有無	なし	参加方法	直接訪問可（予約・連絡不要）<br>＊初回の連絡も不要
SNS	なし		

活動内容		
○ 自由交流	講演会	集団療法
○ 学習・情報収集	SST	○ レクリエーション
心理教育	ペアトレ	その他

備 考

宮城県 ｜ 宮城県発達障害者支援センターえくぼ

発達障害者支援センター　専門家のサポート体制あり

開催場所	仙台市以外の宮城県内公民館等の施設<br>〒981-3213 宮城県仙台市泉区南中山5丁目 2-1		
連絡先	022-376-5306<br>m-ekubo@abelia.ocn.ne.jp	支援先のHP	https://fukushi.miyagi-sfk.net/chuo/chuo-office/ekubo/
参加条件：当事者会	発達障害特性、成人、仙台市以外の宮城県在住の当事者<br>特記すべき条件なし（付き添い）	開催	年に4回／対面
参加条件：家族会	開催なし	参加費	無料
参加条件：診断の有無	なし	参加方法	事前連絡必須（予約・連絡必須）
SNS	なし		

活動内容		
○ 自由交流	○ 講演会	集団療法
○ 学習・情報収集	SST	○ レクリエーション
心理教育	ペアトレ	その他

備 考　全国に同団体あり

宮城県　宮城県自閉症協会

家族会　専門家のサポート体制あり

開催場所	仙台市福祉プラザ等<br>〒984-0816 宮城県仙台市若林区河原町2-2-3　南材ホーム内		
連絡先	080-3328-3802<br>m_autism@yahoo.co.jp	支援先のHP	https://blog.canpan.info/miyagi/
参加条件　当事者会	開催なし	開催	月に2回／対面
参加条件　家族会	宮城県内に居住または拠点を置く当事者と保護者　＊ただし、当事者は発達障害特性	参加費	有料　入会金3000円<br>年会費5000円
参加条件　診断の有無	なし	参加方法	会員制
SNS	なし		
活動内容	○ 自由交流<br>○ 学習・情報収集<br>心理教育	○ 講演会<br>SST<br>ペアトレ	集団療法<br>レクリエーション<br>その他
備 考	全国に同団体あり		

福島県　「大人の発達障害＆HSPの特徴を持つ人の分かち合いの会」特定非営利活動法人Heartis

当事者会　専門家のサポート体制あり

開催場所	福島市café　tetote・郡山市カフェテラス四季<br>〒960-8251　福島県福島市北沢又字上日行壇10-16		
連絡先	080-5382-0221<br>heartis0221@gmail.com	支援先のHP	http://heartis-f.com
参加条件　当事者会	発達障害特性、成人の当事者<br>特記すべき条件なし（付き添い）	開催	月に1回／対面
参加条件　家族会	開催なし	参加費	有料1000円（資料代込）
参加条件　診断の有無	なし	参加方法	事前連絡必須（予約・連絡必須）
SNS	heartis_f		
活動内容	○ 自由交流<br>学習・情報収集<br>心理教育	講演会<br>SST<br>ペアトレ	集団療法<br>レクリエーション<br>その他
備 考			

福島県 ｜ 精神/発達/ひきこもり当事者会 リカバリー福島

当事者会　専門家のサポート体制あり

開催場所		福島市旧御倉邸		
連絡先		koji.okazaki.recovery@gmail.com	支援先のHP	https://kojiokazakijapanto.wixsite.com/recovery-fukushima
参加条件	当事者会	特記すべき条件なし	開催	月に2回／対面、オンライン
	家族会	開催なし	参加費	オンライン：無料<br>対面　　：300円
	診断の有無	なし	参加方法	事前連絡必須（予約・連絡必須）
SNS		X @recovery_fksm　Facebook 精神/発達障がい当事者会『リカバリー福島』		

活動内容

○ 自由交流	○ 講演会	集団療法
○ 学習・情報収集	SST	○ レクリエーション
心理教育	ペアトレ	その他

備考

栃木県　発達障害当事者会リフト

当事者会　専門家のサポート体制あり

開催場所	宇都宮市内の公共施設		
連絡先	adhdasdld@aol.com	支援先のHP	https://adhdasdld.livedoor.blog
参加条件：当事者会	特記すべき条件なし	開催	月に2回／対面、オンライン
参加条件：家族会	開催なし	参加費	茶話会：無料（対面、オンラインともに）<br>ワークショップ：実費
参加条件：診断の有無	なし	参加方法	直接訪問可（予約連絡不要）<br>＊初回の連絡も不要
SNS	X @sFKtpKBtxjtAHg4		

活動内容

○ 自由交流	講演会	集団療法
○ 学習・情報収集（※備考参照）	SST	レクリエーション
心理教育	ペアトレ	その他

備 考

茶話会でのみ参加者との対話が可能（対人トラブル予防のため、連絡先交換制限）
※ 茶話会（薬の効果、ハローワークの活用方法、コミュニケーション技術の情報等）や、
お試しワークショップ（コミュニケーションや優先順位等に関する学習会）

栃木県　「あすカフェ」（株）インクルー

当事者会　専門家のサポート体制あり

開催場所	（株）インクルー<br>〒320-0808　栃木県宇都宮市宮園町8-2　松島ビル2F		
連絡先	028-689-9977<br>info@inclu.co.jp	支援先のHP	https://inclu.co.jp/support/
参加条件：当事者会	コミュニケーションに悩む学生<br>当社の事業所利用者	開催	月に3回／対面
参加条件：家族会	開催なし	参加費	無料
参加条件：診断の有無	なし	参加方法	事前連絡必須（予約・連絡必須）
SNS	X @inclu_info　Facebook inclu.utsunomiya		

活動内容

○ 自由交流	講演会	集団療法
学習・情報収集	○ SST	レクリエーション
心理教育	ペアトレ	○ その他（※備考参照）

備 考

※ 講義メインの生活・仕事に役立つプログラム

栃木県 ｜「おひさまサロン」特定非営利活動法人おひさまクラブ

家族会　専門家のサポート体制あり

開催場所	（おひさまサロン）栃木県栃木市大平地域福祉センター「ふるさとふれあい館」研修室<br>〒328-0075 栃木県栃木市箱森町25-59
連絡先	0282-24-8065<br>hy-saitoh@cc9.ne.jp
支援先のHP	http://ohisama-club.life.coocan.jp
参加条件：当事者会	開催なし
参加条件：家族会	会員の家族（当事者も可）
参加条件：診断の有無	なし
開催	月に1回／対面
参加費	無料
参加方法	直接訪問可（予約・連絡不要）<br>＊初回のみ連絡必須
SNS	なし
活動内容	○ 自由交流、○ 学習・情報収集、○ 心理教育、○ 講演会
備考	初回～数回は会員でなくても参加可能

（活動内容の選択肢：自由交流／学習・情報収集／心理教育／講演会／SST／ペアトレ／集団療法／レクリエーション／その他）

群馬県 ｜「当事者会」サンキャリア

就労移行支援事業所　専門家のサポート体制あり

開催場所	〒370-0069 群馬県高崎市飯塚町149-3
連絡先	070-4028-7543<br>suncareer2023@gmail.com
支援先のHP	https://genuine-llc.com/suncareer/
参加条件：当事者会	18歳以上の方
参加条件：家族会	開催なし
参加条件：診断の有無	なし
開催	不定期（開催しない年もある）／対面
参加費	無料
参加方法	事前連絡必須（予約・連絡必須）
SNS	X @IT0782992386545　＊高崎市の「IT特化型就労移行支援事業所」サンキャリア
活動内容	○ 自由交流、○ 心理教育、○ レクリエーション
備考	

（活動内容の選択肢：自由交流／学習・情報収集／心理教育／講演会／SST／ペアトレ／集団療法／レクリエーション／その他）

関東地方

群馬県　「ひよこむぅん」群馬自助会ぷぅぷぷう

当事者会

開催場所	群馬県社会福祉総合センター		
連絡先	なし	支援先のHP	なし
参加条件：当事者会	発達障害特性、女性の当事者	開催	月に1回／対面
参加条件：家族会	開催なし	参加費	有料300円
参加条件：診断の有無	なし	参加方法	事前連絡必須（予約・連絡必須）
SNS	@hiyokomoonGunma		

活動内容

○ 自由交流	講演会	○ 集団療法
○ 学習・情報収集	SST	レクリエーション
心理教育	ペアトレ	○ その他（※備考参照）

備考

参加人数は6名までに限定（全員が自分の話をできるようにするため）
※ インナーチャイルドワーク、幼少期の記憶を引き起こすトラウマ解消

埼玉県　特定非営利活動法人チャイルド・ギフト

NPO法人　専門家のサポート体制あり

開催場所	川口駅周辺のコワーキングスペース<br>〒332-0035 埼玉県川口市西青木3-10-5-401号		
連絡先	050-3152-2867（自動応答）	支援先のHP	https://childgift.org/
参加条件：当事者会	発達障害特性の当事者<br>当事者の許可が下りた方（付き添い）	開催	月に1回／対面
参加条件：家族会	開催なし	参加費	賛助会員年間3000円<br>（最初の1、2回は無料参加可）
参加条件：診断の有無	なし	参加方法	直接訪問可（予約・連絡不要）<br>＊初回のみ連絡必須
SNS	なし		

活動内容

○ 自由交流	○ 講演会	集団療法
学習・情報収集	○ SST	レクリエーション
心理教育	○ ペアトレ	○ その他（※備考参照）

備考

オンラインは休止中
※ 家族全員参加可能な、チャイルドギフト式ペアトレ

千葉県　ひまわり発達本人の会

当事者会、家族会　専門家のサポート体制あり

開催場所	流山市初石公民館<br>（千葉県流山市西初石）		
連絡先	090-3696-1589	支援先のHP	https://www.kashiwa-yotsuba.jp/himawari.html
参加条件・当事者会	発達障害特性、その他障害の当事者<br>家族、パートナー（付き添い）	開催	月に1回／対面
参加条件・家族会	家族、パートナー（当事者も可）	参加費	会員になると年会費1000円
参加条件・診断の有無	なし	参加方法	直接訪問可（予約・連絡不要）<br>＊初回の連絡も不要
SNS	なし		

活動内容：
- ○ 自由交流
- ○ 学習・情報収集
- 心理教育
- ○ 講演会
- SST
- ペアトレ
- 集団療法
- レクリエーション
- その他

備考：

千葉県　発達障害当事者会「アネモネ」

当事者会

開催場所	市川市市民活動支援センター		
連絡先	なし	支援先のHP	https://ichikawa-anemone.localinfo.jp/
参加条件・当事者会	発達障害特性、成人（※1備考参照）の当事者<br>（付き添いは各当事者１名程）	開催	月に1回／対面
参加条件・家族会	開催なし	参加費	無料
参加条件・診断の有無	なし	参加方法	事前連絡必須（予約・連絡必須）<br>申し込みはこくちーずのみ
SNS	X @ADHD_ANEMONE		

活動内容：
- ○ 自由交流
- ○ 学習・情報収集
- 心理教育
- 講演会
- ○ SST
- ペアトレ
- 集団療法
- レクリエーション
- ○ その他（※2備考参照）

備考：
一人で駅まで来られる方対象、一会が最大14名まで
※1　20～60歳の方
※2　ワークショップ「スモールステップを考えよう」、アイスブレイク

千葉県　凸凹の集い・優しい時間

当事者会　専門家のサポート体制あり

開催場所	オンライン		
連絡先	090-6160-1410<br>asupe.db@gmail.com	支援先のHP	https://ameblo.jp/asupetorisetu/
参加条件：当事者会	発達障害特性の当事者・家族･支援者・どなたでも	開催	月に1回／オンライン（zoom練習付き）
参加条件：家族会	開催なし	参加費	無料
参加条件：診断の有無	なし	参加方法	事前連絡必須（予約・連絡必須）
SNS	なし		

活動内容

- ○ 自由交流
- 学習・情報収集
- 心理教育
- 講演会
- SST
- ペアトレ
- 集団療法
- レクリエーション
- その他

備 考　隔月で午前、週末夜にて開催

東京都　アスペルガー・アラウンド

ピアサポートを活用した支援団体　家族会　専門家のサポート体制あり

開催場所	東京都、静岡県、千葉県、福岡県、オンライン<br>東京都多摩市 他		
連絡先	asperger.around@gmail.com	支援先のHP	http://asperger-around.blog.jp/
参加条件：当事者会	開催なし	開催	月に1回／対面、オンライン
参加条件：家族会	家族、パートナー、支援者<br>＊当事者はカサンドラ状態のみ	参加費	有料（イベントによって異なる）
参加条件：診断の有無	なし	参加方法	事前連絡必須（予約・連絡必須）
SNS	なし		

活動内容

- ○ 自由交流
- ○ 学習・情報収集
- 心理教育
- ○ 講演会
- SST
- ペアトレ
- 集団療法
- レクリエーション
- ○ その他（カサンドラ脱出プログラム、メンター事業）

備 考　精神科受診中の方は主治医と相談の上参加
他県にも同団体あり

東京都 ｜ みどる中高年発達障害当事者会

当事者会

開催場所	首都圏各地		
連絡先	050-3701-5701<br>info@midoru.net	支援先のHP	https://midoru.net
参加条件 当事者会	発達障害特性、40歳以上の当事者<br>特記すべき条件なし（付き添い）	開催	月に1回／対面、オンライン（追加の予定あり）
参加条件 家族会	開催なし	参加費	有料500円
参加条件 診断の有無	なし	参加方法	事前連絡必須（予約・連絡必須）
SNS	X @midoru2014		

活動内容

○ 自由交流	講演会	集団療法
○ 学習・情報収集	SST	レクリエーション
心理教育	ペアトレ	○ その他（情報交換・相互相談）

備 考：非当事者には、別途寄付を依頼

東京都 ｜ あおい発達障害当事者会

当事者会　専門家のサポート体制あり

開催場所	東京都		
連絡先	なし	支援先のHP	https://chofu-npo-supportcenter.jp/circle/?unitid=kus
参加条件 当事者会	発達障害特性の当事者	開催	月に1回／対面
参加条件 家族会	開催なし	参加費	イベントにより様々
参加条件 診断の有無	なし	参加方法	事前連絡必須（予約・連絡必須）
SNS	Facebook あおい発達障害当事者会		

活動内容

○ 自由交流	○ 講演会	集団療法
○ 学習・情報収集	○ SST	○ レクリエーション
○ 心理教育	ペアトレ	その他

備 考：イベントにより参加条件あり

東京都 「ASN」東京都自閉症協会　高機能自閉症・アスペルガー部会

当事者会、 家族会

開催場所	主に杉並区<br>〒170-0005 東京都豊島区南大塚3-43-11福祉財団ビル7F		
連絡先	03-6907-3531<br>autism@bz04.plala.or.jp	支援先のHP	https://autism.jp/
参加条件 当事者会	発達障害特性、成人の当事者	開催	定例会：月に1回／対面<br>当事者会：年に2〜3回／対面
参加条件 家族会	特記すべき条件なし<br>＊ただし当事者は発達障害特性、成人	参加費	東京都自閉症協会会員 無料<br>その他 500円
参加条件 診断の有無	なし	参加方法	事前連絡必須（予約・連絡必須）
SNS	X @tokyoautism　Facebook tojitokyo		

活動内容			
	○ 自由交流	講演会	集団療法
	○ 学習・情報収集	SST	レクリエーション
	心理教育	ペアトレ	その他

備 考　全国に同団体あり

東京都 発達障害を一緒に語る会

当事者会

開催場所	北沢ボランティアビューロー		
連絡先	nyan1202@gmail.com	支援先のHP	http://setahattatsu.wp.xdomain.jp/katarukai
参加条件 当事者会	特記すべき条件なし	開催	月に1回／対面、 オンライン、ハイブリッド
参加条件 家族会	開催なし	参加費	無料
参加条件 診断の有無	なし	参加方法	直接訪問可（予約・連絡不要）<br>＊初回の連絡も不要
SNS	Facebook hattatsukatarukai		

活動内容			
	○ 自由交流	講演会	集団療法
	○ 学習・情報収集	SST	○ レクリエーション
	心理教育	ペアトレ	その他

備 考　会場として「居場所カフェ コモリナ」を使用する場合もあり

東京都 | カラフル@はーと

当事者会

開催場所	東京都中野区にある公共施設の会議室<br>〒164-0001 東京都中野区中野5-68-7 スマイルなかの4F/5F会議室		
連絡先	info@lgbtcath.com	支援先のHP	https://lgbtcath.com
参加条件 当事者会	LGBTQ当事者、成人、メンタルヘルスの問題を抱えている当事者	開催	週に1回／対面
参加条件 家族会	開催なし	参加費	有料200円
参加条件 診断の有無	なし	参加方法	事前連絡必須（予約・連絡必須）
SNS	X @LGBTCatH　Facebook LGBTCatH		

活動内容

○ 自由交流	○ 講演会	集団療法
学習・情報収集	SST	レクリエーション
心理教育	ペアトレ	その他

備考：複数会あり

東京都 | 発達障害当事者座談会

当事者会　専門家のサポート体制あり

開催場所	芝浦オフィス<br>〒108-0023 東京都港区芝浦4-12-31 VORT芝浦WaterFront 6F		
連絡先	03-6809-6985（芝浦オフィス）<br>job_info@happy-terrace.com	支援先のHP	https://decoboco-base.com/
参加条件 当事者会	発達障害特性の当事者、グレーゾーンの方<br>家族（付き添い）	開催	月に2回／対面
参加条件 家族会	開催なし	参加費	無料
参加条件 診断の有無	なし	参加方法	事前連絡必須（予約・連絡必須）
SNS	なし		

活動内容

○ 自由交流	講演会	集団療法
○ 学習・情報収集	SST	レクリエーション
心理教育	ペアトレ	○ その他（個人面談）

備考：
中野オフィス、柏オフィスでも開催あり
イベントの詳細はこくちーずHPで確認
https://www.kokuchpro.com/group/decoboco_base/

東京都　ナマバラ実行委員会

任意団体（ボランティア）

開催場所	世田谷ボランティア協会　会議室		
連絡先	namabara21@gmail.com	支援先のHP	http://setahattatsu.wp.xdomain.jp/namabara
参加条件 当事者会	特記すべき条件なし	開催	2〜3ヶ月に1回／対面
参加条件 家族会	開催なし	参加費	有料200円
参加条件 診断の有無	なし	参加方法	直接訪問可（予約・連絡不要）<br>＊初回の連絡も不要
SNS	namabara2015		

活動内容			
	自由交流	講演会	集団療法
	○ 学習・情報収集	SST	レクリエーション
	心理教育	ペアトレ	その他

備 考

東京都　東京・多摩「大人の発達障害」当事者会

当事者会　専門家のサポート体制あり

開催場所	日野市中央福祉センター		
連絡先	090-9950-2358<br>solalis@t.vodafone.ne.jp	支援先のHP	なし
参加条件 当事者会	発達障害特性、困り感のある成人の当事者<br>特記すべき条件なし（付き添い）	開催	月に2回／対面
参加条件 家族会	開催なし	参加費	有料500円
参加条件 診断の有無	なし	参加方法	事前連絡必須（予約・連絡必須）
SNS	東京・多摩「大人の発達障害」当事者会		

活動内容			
	○ 自由交流	○ 講演会	集団療法
	学習・情報収集	SST	レクリエーション
	心理教育	ペアトレ	○ その他（会話のワークショップ）

備 考

東京都 ADHD交流会

当事者会　専門家のサポート体制あり

開催場所	東京都葛飾区、足立区		
連絡先	info@sunowa.net	支援先のHP	https://sunowa.net/social/
参加条件 当事者会	発達障害特性の当事者<br>特記すべき条件なし（付き添い）	開催	2〜3ヶ月に1回／対面、オンライン
参加条件 家族会	開催なし	参加費	有料2000円
参加条件 診断の有無	なし	参加方法	事前連絡必須（予約・連絡必須）
SNS	なし		

活動内容
- ○ 自由交流
- ○ 学習・情報収集
- ○ 心理教育
- ○ 講演会
- SST
- ペアトレ
- 集団療法
- レクリエーション
- その他

備考

東京都 「ASDグレーゾーンの交流会」発達パートナーズ

当事者会、家族会　専門家のサポート体制あり

開催場所	東京都、埼玉県		
連絡先	hattatsupartners@gmail.com	支援先のHP	https://hattatsupartners.jimdofree.com
参加条件 当事者会	発達障害特性の当事者<br>特記すべき条件なし（付き添い）	開催	月に2回／対面
参加条件 家族会	特記すべき条件なし<br>＊ただし、当事者は発達障害特性	参加費	有料1000円（資料代込）
参加条件 診断の有無	なし	参加方法	事前連絡必須（予約・連絡必須）
SNS	X @tomorrow_gray　ブログ：https://hattatsupartners.com		

活動内容
- ○ 自由交流
- ○ 学習・情報収集
- 心理教育
- 講演会
- SST
- ペアトレ
- 集団療法
- レクリエーション
- その他

備考　神奈川県・埼玉県にも同団体あり

東京都　「DX会、NODE」NPO法人エッジ

当事者会

開催場所	公園など屋外、団体事務所、居酒屋など		
連絡先	なし	支援先のHP	https://www.npo-edge.jp/
参加条件　当事者会	発達障害特性の当事者<br>（当事者以外）研究者、支援者	開催	月に2回、2～3ヶ月に1回／<br>対面、オンライン、ハイブリッド
参加条件　家族会	開催なし	参加費	無料
参加条件　診断の有無	なし	参加方法	事前連絡必須（予約・連絡必須）
SNS	特定非営利活動法人エッジ　NPO EDGE		

活動内容

○ 自由交流	講演会	集団療法
学習・情報収集	SST	レクリエーション
心理教育	ペアトレ	○ その他（※備考参照）

備 考

※ 自分たちの発信について、レクリエーション（屋外の活動）
DX会：当事者がまったりとする会 / NODE：当事者が積極的に発信する会
主にディスレクシアの支援が目的

東京都　いろは会

当事者会

開催場所	都内		
連絡先	irohakai.access@gmail.com	支援先のHP	https://irohakai.jimdofree.com/
参加条件　当事者会	発達障害特性、成人、<br>LGBTs（性的少数者）の当事者	開催	月に1回／対面、オンライン
参加条件　家族会	開催なし	参加費	オンライン：無料<br>対面：有料（0円～1000円程度）※1備考参照
参加条件　診断の有無	なし	参加方法	事前連絡必須（予約・連絡必須）
SNS	@IrohaKai2022		

活動内容

自由交流	講演会	集団療法
学習・情報収集	SST	レクリエーション
心理教育	ペアトレ	○ その他（※2備考参照）

備 考

※1 スペースを使う場合、その額によって変動
※2 時間を人数で割って決めてその人が話したいことを話す。状況によってはフリートーク
半年に一度オフ会を開催。博物館やスペースを借りてゲームなど

東京都　生きづらい子育てピアの会東京

当事者会

開催場所	東京都障害者福祉会館
連絡先	kosodatepia@gmail.com
支援先のHP	https://kosodatepia.wixsite.com/tokyo
参加条件 当事者会	発達障害特性、精神障害、「子育て」に関心がある当事者<br>家族やパートナー（付き添い）
参加条件 家族会	開催なし
参加条件 診断の有無	なし
開催	月に1回／対面、オンライン
参加費	有料300円
参加方法	事前連絡必須（予約・連絡必須）
SNS	X @kosodate_peer

活動内容

○ 自由交流	講演会	集団療法
学習・情報収集	SST	レクリエーション
心理教育	ペアトレ	その他

備 考

神奈川県　一般社団法人 横浜市自閉症協会

家族会

開催場所	横浜市内<br>〒231-0001神奈川県横浜市中区新港2-2-1横浜ワールドポーターズ6F NPOスクエア内
連絡先	045-663-0019<br>HPの問い合わせフォームより
支援先のHP	https://yokohama-yamabiko.org
参加条件 当事者会	開催なし
参加条件 家族会	正会員（当事者も可）
参加条件 診断の有無	なし
開催	活動により月に1回から2～３ヶ月に1回／対面、オンライン
参加費	有料7500円（年間）
参加方法	事前連絡必須の活動あり（予約・連絡必須）
SNS	X @ykhm_yamabiko　Facebook yokohama.yamabiko

活動内容

○ 自由交流	○ 講演会	集団療法
○ 学習・情報収集	SST	○ レクリエーション
心理教育	ペアトレ	その他

備 考　全国に同団体あり

富山県 「はったつスペース」NPO法人和おん

当事者会 専門家のサポート体制あり

開催場所	福祉サービス事業所内<br>〒939-0341富山県射水市三ケ2524-1		
連絡先	0766-95-4063<br>waon-imizu@nifty.com	支援先のHP	https://www.waon-imizu.com
参加条件 当事者会	特記すべき条件なし	開催	月に1回／対面
参加条件 家族会	開催なし	参加費	無料
参加条件 診断の有無	なし	参加方法	事前連絡必須（予約・連絡必須）
SNS	なし		

活動内容
- ○ 自由交流
- 学習・情報収集
- ○ 心理教育
- 講演会
- ○ SST
- ペアトレ
- 集団療法
- レクリエーション
- その他

備 考

富山県 「スペクトラムカフェ」富山県自閉症協会

当事者会 専門家のサポート体制あり

開催場所	やねのうえのガチョウ		
連絡先	076-471-8880<br>toyama.asj@gmail.com	支援先のHP	なし
参加条件 当事者会	特記すべき条件なし	開催	月に1回／ハイブリッド
参加条件 家族会	開催なし	参加費	無料
参加条件 診断の有無	なし	参加方法	直接訪問可（予約・連絡不要）<br>＊初回の連絡も不要
SNS	LINE @947tfdfg　Facebook toyamaautism		

活動内容
- ○ 自由交流
- 学習・情報収集
- 心理教育
- 講演会
- SST
- ペアトレ
- 集団療法
- レクリエーション
- ○ その他（ゲストスピーカーも交えて交流）

備 考　事前に連絡があると、お菓子の用意が容易

富山県 「ハッピーサロン」NPO法人ここらいふ

当事者会、家族会 / 専門家のサポート体制あり

開催場所	富山市サンシップとやま（水曜日）、サンフォルテ富山（土曜日）		
連絡先	080-6369-0612<br>info@coco-life.jp	支援先のHP	http://inari.fool.jp/cocolife/sample-page/
参加条件：当事者会	特記すべき条件なし	開催	週に1回／基本的に対面<br>＊ハイブリッドやオンラインのみの時あり
参加条件：家族会	特記すべき条件なし（当事者も可）	参加費	無料、 個別相談は500円
参加条件：診断の有無	なし	参加方法	直接訪問可（予約・連絡不要）<br>＊初回の連絡も不要、 個別相談は予約あり
SNS	cocolife.toyama		

活動内容

○ 自由交流	講演会	集団療法
○ 学習・情報収集	○ SST	○ レクリエーション
○ 心理教育	ペアトレ	○ その他（個別相談）

備 考　開催日、場所等はホームページ・facebookにて要確認（変更する場合があるため）

富山県 一般社団法人Ponteとやま

居場所・カフェ / 専門家のサポート体制あり

開催場所	みやの森カフェ、シェアハウスLibero<br>〒939-1406 富山県砺波市宮森303		
連絡先	0763-77-3733<br>miyanomori.ponte@gmail.com	支援先のHP	https://ponte-toyama.com/
参加条件：当事者会	特記すべき条件なし	開催	毎日（※備考参照） ／対面、オンライン
参加条件：家族会	開催なし	参加費	若者当事者会：無料<br>相談、その他の活動：有料
参加条件：診断の有無	なし	参加方法	事前連絡必須（予約・連絡必須）
SNS	X @PonteMiyanomori　Instagram ponte.miyanomori　Facebook miyanomori.ponte		

活動内容

○ 自由交流	○ 講演会	集団療法
学習・情報収集	SST	○ レクリエーション
○ 心理教育	ペアトレ	○ その他（就労サポート・学習サポート）

備 考　複数事業実施　※木～土…みやの森カフェ
月～水、金…子ども・若者の居場所活動
土日…プログラム，相談会
その他当事者会は随時

富山県　NPO法人はぁとぴあ21

当事者会、 家族会

開催場所	はぁとぴあ21の施設<br>〒939-0341 富山県射水市三ヶ2467		
連絡先	0766-75-3885<br>heartopia21@gmail.com	支援先のHP	https://www.heartopia21.com/
参加条件 当事者会	特記すべき条件なし	開催	毎日（月～土）／ハイブリッド
参加条件 家族会	特記すべき条件なし（当事者も可）	参加費	当事者：無料、当事者以外：500円
参加条件 診断の有無	なし	参加方法	直接訪問可（予約・連絡不要）<br>＊初回の連絡も不要
SNS	なし		

活動内容			
	○ 自由交流	○ 講演会	集団療法
	○ 学習・情報収集	○ SST	○ レクリエーション
	心理教育	ペアトレ	○ その他（作品展、音楽鑑賞会、遠足）

備 考　複数会あり　親の会（親）、女子会・男子会（子）など

富山県　ふらっとサロン

家族会

開催場所	まちなかサロン（氷見市）		
連絡先	090-2098-2296	支援先のHP	なし
参加条件 当事者会	開催なし	開催	2～3ヶ月に1回 ／対面
参加条件 家族会	特記すべき条件なし<br>＊ただし、当事者は発達障害特性	参加費	有料200円（お茶代）
参加条件 診断の有無	なし	参加方法	事前連絡必須（予約・連絡必須）
SNS	なし		

活動内容			
	○ 自由交流	○ 講演会	集団療法
	○ 学習・情報収集	SST	レクリエーション
	心理教育	○ ペアトレ	その他

備 考

石川県　大人の発達障害を明るく語る会「ここらぼ」

当事者会　専門家のサポート体制あり

開催場所	シェアマインド金沢<br>〒920-8204 石川県金沢市戸水1-25		
連絡先	080-5853-0310<br>beyond.y.mizuto@gmail.com	支援先のHP	なし
参加条件　当事者会	発達障害特性、成人の当事者<br>特記すべき条件なし（付き添い）	開催	月に1回／対面、オンライン
参加条件　家族会	開催なし	参加費	有料500円
参加条件　診断の有無	なし	参加方法	事前連絡必須（予約・連絡必須）
SNS	cocorolabo		

活動内容

○ 自由交流	○ 講演会	集団療法
○ 学習・情報収集	SST	レクリエーション
○ 心理教育	ペアトレ	○ その他（BBQ）

備考

石川県　「シニア部、サークル活動フロンティア」特定非営利活動法人アスペの会石川

当事者会、家族会　専門家のサポート体制あり

開催場所	当会事務所、レストラン、カラオケ、美術館等<br>〒920-0865 石川県金沢市長町1-4-11		
連絡先	kanazawa-asupe@abeam.ocn.ne.jp	支援先のHP	https://aspe.sub.jp/
参加条件　当事者会	発達障害特性<br>家族が会の運営に協力できる方	開催	月に3回／基本的には対面<br>＊状況によってオンラインあり
参加条件　家族会	利用者の家族<br>＊ただし、当事者は発達障害特性	参加費	有料2000円
参加条件　診断の有無	あり	参加方法	事前連絡必須（予約・連絡必須）<br>家族の会員（入会すること）が参加条件
SNS	準備中		

活動内容

○ 自由交流	講演会	集団療法
学習・情報収集	○ SST	レクリエーション
心理教育	ペアトレ	○ その他（※備考参照）

備考

複数会あり、HP参照
※ レクリエーション（作業的な内容ではない）、余暇支援

石川県　金沢大学子どものこころの発達研究センター 自閉症サイエンスカフェ「カフェで語ろう!!!自閉症」

サイエンスカフェ　専門家のサポート体制あり

開催場所	しいのき迎賓館<br>〒920-0962 石川県金沢市広坂2-1-1		
連絡先	076-234-4213<br>childdev@med.kanazawa-u.ac.jp	支援先のHP	https://kodomokokoro.w3.kanazawa-u.ac.jp/event/cafe.html
参加条件：当事者会	特記すべき条件なし	開催	奇数月22日／対面<br>（会場の都合で日程変更の場合あり）
参加条件：家族会	開催なし	参加費	無料
参加条件：診断の有無	なし	参加方法	直接訪問可（予約・連絡不要）<br>＊初回の連絡も不要
SNS	なし		

活動内容

- ○ 自由交流
- 学習・情報収集
- 心理教育
- 講演会
- SST
- ペアトレ
- 集団療法
- レクリエーション
- その他

備 考：自閉症に関心のある者が集い、語り合うカフェ。当事者や家族以外に、地域の方どなたも自由に立ち寄り可

石川県　金沢「当事者研究」研究会

学習会

開催場所	金沢市松ヶ枝福祉館		
連絡先	090-3450-7749<br>oriteiku.kanazawa@gmail.com	支援先のHP	なし
参加条件：当事者会	特記すべき条件なし	開催	月に2回／ハイブリッド
参加条件：家族会	開催なし	参加費	無料（資料代がかかる場合もあり）
参加条件：診断の有無	なし	参加方法	直接訪問可（予約・連絡不要）<br>＊初回のみ連絡必須
SNS	リカバリーかなざわ		

活動内容

- 自由交流
- ○ 学習・情報収集
- 心理教育
- 講演会
- ○ SST
- ペアトレ
- 集団療法
- レクリエーション
- その他

備 考：

石川県 ｜ 石川県自閉症協会

家族会　専門家のサポート体制あり

開催場所	主に金沢市内		
連絡先	076-257-1327<br>mi09te27kigoshi@yahoo.co.jp	支援先のHP	なし
参加条件 当事者会	開催なし	開催	不定期（開催しない年もある）／対面
参加条件 家族会	特記すべき条件なし（当事者も可）	参加費	無料
参加条件 診断の有無	なし	参加方法	事前連絡必須（予約・連絡必須）
SNS	なし		

活動内容
- ○ 自由交流
- ○ 学習・情報収集
- 心理教育
- ○ 講演会
- SST
- ペアトレ
- 集団療法
- レクリエーション
- その他

備考：全国に同団体あり

福井県 ｜ 「AOZORA倶楽部」特定非営利活動法人 AOZORA福井

当事者会　専門家のサポート体制あり

開催場所	AOZORA<br>〒919-0445 福井県坂井市春江町中筋16-10-2		
連絡先	0776-63-6098<br>abogadokanri@yahoo.co.jp	支援先のHP	http://aozorafukui.jp/index.html
参加条件 当事者会	口づてで紹介されて事前アセスメントを受けた当事者<br>特記すべき条件なし（付き添い）	開催	不定期（開催しない年もある）／対面
参加条件 家族会	開催なし	参加費	無料
参加条件 診断の有無	なし	参加方法	事前連絡必須（予約・連絡必須）
SNS	なし		

活動内容
- ○ 自由交流
- 学習・情報収集
- 心理教育
- 講演会
- SST
- ペアトレ
- 集団療法
- レクリエーション
- その他

備考：

福井県 ｜「ここから」一般社団法人今ここ

家族会　専門家のサポート体制あり

開催場所	今ここ<br>〒918-8105 福井県福井市木田3-3113-2		
連絡先	0776-63-6859<br>imakoko@imakoko.or.jp	支援先のHP	https://kokomariko.com/
参加条件 当事者会	開催なし	開催	月に1回／対面、オンライン
参加条件 家族会	利用者の家族<br>＊ただし、当事者は発達障害特性	参加費	無料
参加条件 診断の有無	なし	参加方法	事前連絡必須（予約・連絡必須）
SNS	imakoko.or.jp		

活動内容

自由交流	講演会	集団療法
学習・情報収集	SST	レクリエーション
心理教育	○ ペアトレ	○ その他（個人面談）

備考　生きづらさを抱えた方が利用しているフリースペース（当事者会）休止中

長野県 ｜ げんき会

当事者会　専門家のサポート体制あり

開催場所	あいとぴあ臼田<br>〒384-0414 長野県佐久市下越16-5		
連絡先	090-4905-9748<br>takoyaki2116633@icloud.com	支援先のHP	なし
参加条件 当事者会	特記すべき条件なし	開催	月に1回／ハイブリッド
参加条件 家族会	開催なし	参加費	基本的に無料。内容によって参加費あり<br>（例：講師代一人当たり500円）
参加条件 診断の有無	なし	参加方法	直接訪問可（予約・連絡不要）<br>＊初回の連絡も不要
SNS	genkikai_saku　松本美恵子　（ミエサン）		

活動内容

○ 自由交流	○ 講演会	集団療法
○ 学習・情報収集	○ SST	○ レクリエーション
○ 心理教育	ペアトレ	○ その他（専門医によるワークショップ）

備考

岐阜県 「ぴあサラダ」飛騨圏域発達障がい支援センターそらいろ

当事者会　専門家のサポート体制あり

開催場所	福祉センター、福祉事業所内交流スペース、民間交流スペース<br>〒506-0058 岐阜県高山市山田町831-43		
連絡先	057-735-6780<br>sorairo@hida-jikoukai.or.jp	支援先のHP	なし
参加条件 当事者会	発達障害特性、成人の当事者	開催	月に1回／ハイブリッド
参加条件 家族会	開催なし	参加費	無料、 寄付金（任意）
参加条件 診断の有無	なし	参加方法	直接訪問可（予約・連絡不要）<br>＊初回の連絡も不要
SNS	hida_sorairo		

活動内容

○ 自由交流	講演会	集団療法
学習・情報収集	SST	○ レクリエーション
心理教育	ペアトレ	その他

備 考

岐阜県 「ぴあっと」岐阜県発達障害者支援センター

当事者会　専門家のサポート体制あり

開催場所	岐阜県発達障害者支援センター<br>〒502-0854 岐阜県岐阜市鷺山向井2563-18		
連絡先	058-233-5116	支援先のHP	https://www.pref.gifu.lg.jp/page/20822.html
参加条件 当事者会	発達障害特性、成人、 地域限定の当事者	開催	月に1回／ハイブリッド
参加条件 家族会	開催なし	参加費	無料
参加条件 診断の有無	なし	参加方法	事前連絡必須（予約・連絡必須）
SNS	なし		

活動内容

○ 自由交流	講演会	集団療法
学習・情報収集	SST	○ レクリエーション
心理教育	ペアトレ	その他

備 考

家族のための交流会もあり。要HP参照。
「Dear たんぽぽ」（概ね18歳未満の発達障がい orその疑いのあるお子さんの保護者の方が対象）
「Dear わたげ」　（概ね18歳以上の発達障がい or その疑いのあるお子さんの保護者の方が対象）

愛知県 「当事者会」一般社団法人あいち発達障害サポートネットワーク

当事者会

開催場所	こねっこ弐番館<br>〒466-0815 愛知県名古屋市昭和区山手通５-32 月東ビル１階
連絡先	なし
支援先のHP	https://co-necco.xii.jp
参加条件 当事者会	成人
参加条件 家族会	開催なし
参加条件 診断の有無	なし
開催	月に3回／対面
参加費	有料　お母さんの会1000円<br>女性の会（当事者会）500円
参加方法	事前連絡必須（予約・連絡必須）
SNS	X @coneccosan

活動内容

○ 自由交流	講演会	集団療法
学習・情報収集	SST	レクリエーション
心理教育	ペアトレ	その他

備考：複数会あり。女性の会、お母さんの会など

愛知県 特定非営利法人アスペ・エルデの会

専門家と親の会を合体させた法人　専門家のサポート体制あり

開催場所	各地域の公的な施設<br>〒474-0073 愛知県大府市東新町2－222
連絡先	info@as-japan.jp
支援先のHP	www.as-japan.jp
参加条件 当事者会	発達障害特性、事業所等に通う当事者<br>家族（付き添い）
参加条件 家族会	開催なし
参加条件 診断の有無	あり
開催	2～3ヶ月に1回 ／対面、オンライン
参加費	有料<br>（イベントによって異なるため事前確認必要）
参加方法	事前連絡必須（予約・連絡必須）
SNS	なし

活動内容

○ 自由交流	○ 講演会	集団療法
○ 学習・情報収集	SST	レクリエーション
心理教育	○ ペアトレ	その他

備考：家族会複数あり
岐阜県・三重県にも同団体あり

滋賀県　「大人の発達凹凸の会iroiro~イロイロ~」一般社団法人異才ネットワーク

当事者会　専門家のサポート体制あり

開催場所	大津オルタナティブスクール　トライアンフ<br>〒520-0047 滋賀県大津市浜大津4丁目3-25		
連絡先	090-7885-6301<br>isai_nw@outlook.jp	支援先のHP	https://www.facebook.com/isai.iroiro
参加条件：当事者会	発達障害特性、成人の当事者<br>傍聴のみ可（付き添い）	開催	2～3ヶ月に1回 ／対面
参加条件：家族会	開催なし	参加費	有料500円
参加条件：診断の有無	なし	参加方法	事前連絡必須（予約・連絡必須）
SNS	isai.iroiro		

活動内容

○ 自由交流	講演会	集団療法
○ 学習・情報収集	SST	レクリエーション
心理教育	ペアトレ	その他

備 考

京都府　「Kirala（レクリエーションサークル）」一般社団法人京都府自閉症協会

家族会　専門家のサポート体制あり

開催場所	青少年活動センター<br>〒604-8804 京都府中京区壬生坊城町48-6　京都社会福祉会館３階		
連絡先	075-813-5156<br>askyoto@shirt.ocn.ne.jp	支援先のHP	https://as-kyoto.com/
参加条件：当事者会	開催なし	開催	月に1回 ／対面
参加条件：家族会	特記すべき条件なし（当事者も可）	参加費	有料5000円（年7回活動）
参加条件：診断の有無	なし	参加方法	レクのサークルはメンバー制
SNS	X @ as_kyoto　asd.kyoto		

活動内容

自由交流	講演会	集団療法
学習・情報収集	SST	レクリエーション
心理教育	ペアトレ	○ その他（音楽やダンスなど）

備 考

全国に同団体あり
当事者会は現在準備中

大阪府 グループそのまま

当事者会　専門家のサポート体制あり

開催場所	大阪市内		
連絡先	gsonomama@gmail.com	支援先のHP	https://gsonomama.com
参加条件 当事者会	発達障害特性、成人の当事者<br>特記すべき条件なし（付き添いの人数制限あり）	開催	2〜3ヶ月に1回 ／対面、オンライン
参加条件 家族会	開催なし	参加費	有料1000円（大阪BASIC）
参加条件 診断の有無	なし	参加方法	事前連絡必須（予約・連絡必須）
SNS	X @gsonomama_jp		

活動内容
- ○ 自由交流
- 学習・情報収集
- 心理教育
- 講演会
- SST
- ペアトレ
- 集団療法
- レクリエーション
- ○ その他（※備考参照）

備 考：※ エンカウンターグループをベースにフリートーク

大阪府 さかいハッタツ友の会

当事者会

開催場所	各会により異なる（要ブログ参照）		
連絡先	090-6903-6060<br>duaed021@yahoo.co.jp	支援先のHP	https://sakai-dd.hatenablog.com/
参加条件 当事者会	発達障害特性、成人の当事者<br>特記すべき条件なし（付き添い）	開催	毎日どこかで開催／方法は会によって異なる
参加条件 家族会	開催なし	参加費	無料〜500円まで
参加条件 診断の有無	なし	参加方法	対面：直接訪問可（予約・連絡不要）<br>オンライン：事前連絡必須（予約・連絡必須）
SNS	なし		

活動内容
- ○ 自由交流
- ○ 学習・情報収集
- 心理教育
- 講演会
- SST
- ペアトレ
- ○ 集団療法
- ○ レクリエーション
- その他

備 考：
複数の県に同団体あり（青森県、宮城県、福島県、茨城県、栃木県、群馬県、千葉県、東京都、神奈川県、長野県、新潟県、岐阜県、静岡県、愛知県、三重県、京都府、兵庫県、和歌山県、香川県、福岡県）
各会によって実施日時や条件が異なるため要確認

大阪府 「関西ほっとサロン、北摂ほっとサロン、家族のための相談会」 NPO法人DDAC

当事者会、 家族会

開催場所	芦屋市、茨木市		
連絡先	問い合わせフォームより	支援先のHP	https://www.adhd-west.net/
参加条件 当事者会	発達障害特性、成人の当事者	開催	月に1回、2〜3ヶ月に1回 ／対面
参加条件 家族会	家族、パートナー（当事者不可）	参加費	有料 DDAC会員300円、一般800円
参加条件 診断の有無	なし	参加方法	直接訪問可（予約・連絡不要）<br>＊初回の連絡も不要
SNS	X @adhdwest		

活動内容

○ 自由交流	講演会	集団療法
学習・情報収集	SST	レクリエーション
心理教育	ペアトレ	その他

備 考

兵庫県にも同団体あり
複数会あり

大阪府 「発達障がいについて考える会『カラフル』」 社会福祉法人大阪市城東区社会福祉協議会

当事者会　専門家のサポート体制あり

開催場所	城東区在宅サービスセンターゆうゆう<br>〒536-0005 大阪府大阪市城東区中央2-11-16		
連絡先	06-6936-1153	支援先のHP	https://jyotan-sky1.jimdo.com/
参加条件 当事者会	発達障害特性の当事者<br>特記すべき条件なし（付き添い）	開催	月に1回／対面
参加条件 家族会	開催なし	参加費	無料
参加条件 診断の有無	なし	参加方法	事前連絡推奨
SNS	なし		

活動内容

○ 自由交流	講演会	集団療法
学習・情報収集	SST	レクリエーション
心理教育	ペアトレ	その他

備 考

親の会あり、HP参照

近畿地方

大阪府　阪大自閉症スペクトラム（ASD）カフェ 勤労中高年ASDカフェ

サイエンスカフェ　専門家のサポート体制あり

開催場所	大阪大学		
連絡先	なし	支援先のHP	https://doll2014-cafe.wixsite.com/asdsciencecafe
参加条件：当事者会	発達障害特性、成人の当事者<br>特記すべき条件なし（付き添い）	開催	2〜3ヶ月に1回 ／対面
参加条件：家族会	開催なし	参加費	無料
参加条件：診断の有無	なし	参加方法	事前連絡必須（予約・連絡必須）
SNS	なし		

活動内容			
	○ 自由交流	講演会	集団療法
	○ 学習・情報収集	SST	レクリエーション
	心理教育	ペアトレ	その他

備 考

大阪府　「UnBalanceサロン」成人発達障害当事者自助グループUnBalance

当事者会

開催場所	大阪市平野区		
連絡先	info@unbalance.main.jp	支援先のHP	http://unbalance.main.jp
参加条件：当事者会	特記すべき条件なし	開催	2ヶ月に1回 ／対面
参加条件：家族会	開催なし	参加費	有料500円<br>（年に一度の周年記念講演会時のみ1000円）
参加条件：診断の有無	なし	参加方法	直接訪問可（予約・連絡不要）<br>＊初回の連絡も不要
SNS	unbalance.main.jp		

活動内容			
	○ 自由交流	○ 講演会	集団療法
	学習・情報収集	SST	レクリエーション
	心理教育	ペアトレ	その他

備 考　複数の県に同団体あり（兵庫県、奈良県、岡山県、広島県、熊本県、愛知県、富山県）

大阪府　ゆずの会　大人の発達凸凹自助グループ

当事者会　専門家のサポート体制あり

開催場所	公共の貸会議室		
連絡先	なし	支援先のHP	なし
参加条件：当事者会	発達障害特性、成人の当事者<br>特記すべき条件なし（付き添い）	開催	月に1回 ／対面、オンライン
参加条件：家族会	開催なし	参加費	オンライン：無料、対面：有料300円
参加条件：診断の有無	なし	参加方法	直接訪問可（予約・連絡不要）<br>＊初回の連絡も不要
SNS	(Facebook) ゆずの会大人の発達凹凸自助グループ		

活動内容

○ 自由交流	講演会	集団療法
学習・情報収集	SST	レクリエーション
心理教育	ペアトレ	その他

備 考

兵庫県　はりまADDM 大人の発達自助会

当事者会

開催場所	加古川市、西脇市、淡路島、姫路市など		
連絡先	harimaaddm@gmail.com	支援先のHP	http://www.harima-addm.com/
参加条件：当事者会	特記すべき条件なし	開催	月に1～2回 ／対面、オンライン
参加条件：家族会	開催なし	参加費	有料（HP参照）
参加条件：診断の有無	なし	参加方法	事前連絡推奨（予約・連絡推奨）<br>会場で直接申込も可
SNS	(X) @harimaaddm		

活動内容

○ 自由交流	○ 講演会	集団療法
学習・情報収集	SST	レクリエーション
心理教育	ペアトレ	その他

備 考

兵庫県 「神戸発達お散歩会」神戸のとまり木

当事者会

開催場所	神戸市内の文化センター		
連絡先	totuteam2019@gmail.com	支援先のHP	https://kobebbnet.jimdofree.com/
参加条件 当事者会	発達障害特性、知的障害の当事者<br>特記すべき条件なし（付き添い）	開催	2〜3ヶ月に1回 ／対面
参加条件 家族会	開催なし	参加費	有料500円
参加条件 診断の有無	なし	参加方法	事前連絡必須（予約・連絡必須）
SNS	なし		

活動内容

○ 自由交流	講演会	集団療法
学習・情報収集	SST	レクリエーション
心理教育	ペアトレ	○ その他（散歩を使ったアクティビティ）

備 考

兵庫県 NPO法人ピュアコスモ

当事者会、家族会　専門家のサポート体制あり

開催場所	主に神戸市青少年会館		
連絡先	npopurec@gmail.com	支援先のHP	http://purecosmo.com
参加条件 当事者会	発達障害特性、15歳以上の当事者	開催	当事者交流会　奇数月に1回／対面<br>（必要に応じてオンライン）
参加条件 家族会	発達障害児・者の家族・支援者など<br>（当事者も可）	参加費	無料（一部実費の場合あり）
参加条件 診断の有無	あり（当事者会のみ）	参加方法	インターネットで入会（要入会金・会費）
SNS	なし		

活動内容

○ 自由交流（専門家ボランティア同席）	○ 講演会	集団療法
○ 学習・情報収集	SST	○ レクリエーション
心理教育	ペアトレ	その他

備 考

兵庫県 ｜ 兵庫県自閉症協会

家族会　専門家のサポート体制あり

開催場所	明石サービスセンター西館<br>〒660-0083 兵庫県尼崎市道意町6-2-107　兵庫県自閉症協会事務局		
連絡先	06-6418-4480<br>heg54603@hcc5.bai.ne.jp	支援先のHP	（一社）日本自閉症協会を参照
参加条件：当事者会	開催なし	開催	2～3ヶ月に1回／対面
参加条件：家族会	特記すべき条件なし（当事者も可）	参加費	無料
参加条件：診断の有無	なし	参加方法	事前連絡必須（予約・連絡必須）
SNS	なし		

活動内容：
- ○ 自由交流
- ○ 学習・情報収集
- 心理教育
- ○ 講演会
- SST
- ○ ペアトレ
- 集団療法
- ○ レクリエーション
- その他

備考：全国に同団体あり

兵庫県 ｜ 神戸市自閉症協会

家族会

開催場所	〒651-2117 兵庫県神戸市西区北別府5-9-8 久保千明方 神戸市自閉症協会		
連絡先	078-975-3601	支援先のHP	なし
参加条件：当事者会	開催なし	開催	2～3ヶ月に1回 ／対面
参加条件：家族会	特記すべき条件なし<br>＊ただし、当事者は発達障害特性、親子で参加	参加費	無料、 有料500～3000円<br>（イベントごとに異なる）
参加条件：診断の有無	なし	参加方法	入数制限のある場合もあり
SNS	なし		

活動内容：
- ○ 自由交流
- 学習・情報収集
- 心理教育
- 講演会
- SST
- ペアトレ
- 集団療法
- ○ レクリエーション
- その他

備考：全国に同団体あり

和歌山県 | 特定非営利活動法人　和歌山県自閉症協会

当事者会、 家族会 / 専門家のサポート体制あり

開催場所	開催場所は、その都度協議して決定<br>〒646-0013 和歌山県田辺市南新万13－4（事務所）		
連絡先	0739-25-1018<br>ohkubo@vm.aikis.or.jp	支援先のHP	https://asw.yu-yake.com/
参加条件 当事者会	会員資格を有する当事者<br>特記すべき条件なし（付き添い）	開催	不定期（開催しない年もある）／対面
参加条件 家族会	特記すべき条件なし（当事者も可）	参加費	入会についてはHP参照<br>イベントはその都度検討
参加条件 診断の有無	なし	参加方法	事前連絡必須（予約・連絡必須）<br>電話 or インターネットにて
SNS	なし		

活動内容			
	自由交流	○ 講演会	○ 集団療法
	○ 学習・情報収集	SST	○ レクリエーション
	心理教育	ペアトレ	その他

備 考

全国に同団体あり
当会内部に専門家部会と地域分会あり
家族会あり、HP参照

三重県 | にじいろ　津

当事者会、 家族会

開催場所	なし		
連絡先	なし	支援先のHP	https://www.kokuchpro.com/group/tsu4hattatsu/
参加条件 当事者会	特記すべき条件なし	開催	月に1回 ／対面
参加条件 家族会	特記すべき条件なし（当事者も可）	参加費	有料100円
参加条件 診断の有無	なし	参加方法	直接訪問可（予約・連絡不要）<br>＊初回の連絡も不要
SNS	X @tsushihattatsu		

活動内容			
	○ 自由交流	講演会	集団療法
	○ 学習・情報収集	○ SST	レクリエーション
	心理教育	ペアトレ	その他

備 考

鳥取県　居い場所田園　はなし場

当事者会、家族会　専門家のサポート体制あり

開催場所	地域（まち）でくらす会の施設<br>〒683-0816 鳥取県米子市西倉吉町83-3		
連絡先	0859-35-5647<br>machikura@eagle.ocn.ne.jp	支援先のHP	http://www.machikura.com/seikatsushien-machikura.html
参加条件 当事者会	特記すべき条件なし（付き添い不可）	開催	フリースペース話し場　週に1回／対面<br>カレーを食べる会　月に2回／対面
参加条件 家族会	特記すべき条件なし（当事者不可）	参加費	基本無料<br>月2回のカレーを食べる会のみ材料費300円
参加条件 診断の有無	なし	参加方法	事前連絡必須（予約・連絡必須）
SNS	Facebook 居い場所田園　Instagram @iibasyo_denen		
活動内容	○ 自由交流<br>○ 学習・情報収集<br>心理教育	○ 講演会<br>SST<br>ペアトレ	集団療法<br>○ レクリエーション<br>その他
備 考			

鳥取県　鳥取県自閉症協会

家族会　専門家のサポート体制あり

開催場所	事務局・公共施設<br>〒680-0821 鳥取県鳥取市瓦町601		
連絡先	0857-30-2776<br>asj-tottori@trad.ocn.ne.jp	支援先のHP	https://asj-tt.com/
参加条件 当事者会	開催なし	開催	月に1回、地区ごとに月に１～2回開催／対面、ハイブリッド
参加条件 家族会	特記すべき条件なし<br>＊ただし、当事者は発達障害特性	参加費	有料200円
参加条件 診断の有無	なし	参加方法	直接訪問可（予約・連絡不要）<br>＊初回の連絡も不要<br>オンラインは事前連絡必須（予約・連絡必須）
SNS	なし		
活動内容	○ 自由交流<br>○ 学習・情報収集<br>心理教育	○ 講演会<br>SST<br>ペアトレ	集団療法<br>○ レクリエーション<br>その他
備 考	全国に同団体あり<br>公益事業・委託事業・啓発のための研修会		

島根県 ｜ 山陰発達障害当事者会スモステの会

当事者会　専門家のサポート体制あり

開催場所	松江市内		
連絡先	sumosute_shimane@yahoo.co.jp	支援先のHP	https://jetstarman.jimdofree.com/
参加条件：当事者会	発達障害特性、会の趣旨に賛同された当事者<br>特記すべき条件なし（付き添い）	開催	月に1回／対面
参加条件：家族会	開催なし	参加費	有料300円
参加条件：診断の有無	なし	参加方法	直接訪問可（予約・連絡不要）<br>＊初回のみ連絡必須
SNS	なし		

活動内容		
○ 自由交流	講演会	集団療法
○ 学習・情報収集	SST	レクリエーション
心理教育	ペアトレ	その他

備 考

岡山県 ｜ 岡山県LD等発達障害親の会はあとりんく

家族会　専門家のサポート体制あり

開催場所	有料の貸会場（国際交流センター・岡山県総合福祉・ボランティア・NPO会館「きらめきプラザ」）<br>公民館		
連絡先	okayama.h.link@gmail.com	支援先のHP	http://heartlink-okayama.jimdo.com/
参加条件：当事者会	開催なし	開催	月に1回 ／ハイブリッド
参加条件：家族会	特記すべき条件なし（当事者も可）	参加費	イベントにより様々
参加条件：診断の有無	なし	参加方法	直接訪問可（予約・連絡不要）<br>＊初回の連絡も不要
SNS	heartlink.okayama		

活動内容		
○ 自由交流	○ 講演会	集団療法
○ 学習・情報収集	○ SST	○ レクリエーション
心理教育	ペアトレ	その他

備 考

中国地方

岡山県 「なかまぁず」津山手をつなぐ親の会

本人の会　家族会

開催場所	公民館、福祉会館など		
連絡先	oyanokai_tsuyama@yahoo.co.jp	支援先のHP	なし
参加条件 当事者会	発達障害特性、知的障害の当事者<br>特記すべき条件なし（付き添い）	開催	月に1回／対面
参加条件 家族会	特記すべき条件なし<br>＊ただし、当事者は発達障害特性、知的障害	参加費	有料2000円（年間）
参加条件 診断の有無	なし	参加方法	事前連絡必須（予約・連絡必須）
SNS	oyanokaitsuyama		

活動内容	○ 自由交流<br>○ 学習・情報収集<br>心理教育	○ 講演会<br>SST<br>○ ペアトレ	集団療法<br>○ レクリエーション<br>○ その他（スポーツ、アート活動）

備 考

広島県 「当事者による当事者の為のピアの会」発達障害自助グループ ぽっぽカフェ

当事者会

開催場所	〒731-4312 広島県安芸郡坂町平成ヶ浜1丁目3-19		
連絡先	poppo_cafe_toujisha-0409@yahoo.co.jp	支援先のHP	https://poppo-cafe.site/
参加条件 当事者会	発達障害特性、成人の当事者<br>特記すべき条件なし（付き添い）	開催	月に1回 ／対面、オンライン
参加条件 家族会	開催なし	参加費	有料300円
参加条件 診断の有無	なし	参加方法	直接訪問可（予約・連絡不要）<br>＊初回の連絡も不要
SNS	なし		

活動内容	○ 自由交流<br>学習・情報収集<br>心理教育	講演会<br>SST<br>ペアトレ	集団療法<br>レクリエーション<br>その他

備 考

広島県 ｜「クローバーの広場」一般社団法人クローバーの会

当事者会、 家族会 ｜ 専門家のサポート体制あり

開催場所	公民館等<br>〒734-0015 広島県広島市南区宇品御幸3丁目9-9 1F		
連絡先	070-8833-0968<br>clover2003.hiroshima@gmail.com	支援先のHP	https://hiroshima-clover.com/
参加条件：当事者会	発達障害特性の当事者<br>家族、関係者（付き添い）	開催	月に3回／対面、オンライン
参加条件：家族会	特記すべき条件なし<br>＊ただし、当事者は発達障害特性	参加費	イベントにより様々
参加条件：診断の有無	なし	参加方法	事前連絡必須（予約・連絡必須）
SNS	なし		

活動内容

○ 自由交流	講演会	○ 集団療法
○ 学習・情報収集	SST	レクリエーション
心理教育	ペアトレ	その他

備 考

広島県 ｜ 発達障がいを持つ女性の会「なないろサタデー」

当事者会 ｜ 専門家のサポート体制あり

開催場所	就労継続支援B型事業所　なないろ２階<br>〒720-0061広島県福山市丸之内1-3-9（就労継続支援B型事業所なないろ内）		
連絡先	090-8609-1121<br>nnanairo0@gmail.com	支援先のHP	http://jiyukan.org/archives/2817/
参加条件：当事者会	発達障害特性、成人女性の当事者	開催	週に1回／対面、電話相談
参加条件：家族会	開催なし	参加費	無料
参加条件：診断の有無	あり	参加方法	直接訪問可（予約・連絡不要）<br>＊初回のみ連絡必須
SNS	maikka_nanairo　ブログ：https://ameblo.jp/maikka-nanairo/		

活動内容

○ 自由交流	○ 講演会	集団療法
○ 学習・情報収集	SST	レクリエーション
○ 心理教育	ペアトレ	その他

備 考

徳島県　「クローバー」「カンガルークラブ」NPO法人オーティの会

当事者会、家族会　専門家のサポート体制あり

開催場所		徳島県内		
連絡先		080-6399-1593<br>auti@auti.jp	支援先のHP	http://auti.jp/
参加条件	当事者会	発達障害特性、成人の当事者<br>特記すべき条件なし（付き添い）	開催	月に2回、 キャンプ、バス遠足あり／対面
	家族会	発達障害の診断のある子どもと家族	参加費	有料　クローバー 500円<br>カンガルークラブ 2500円
	診断の有無	あり（カンガルークラブのみ）	参加方法	直接訪問可（予約・連絡不要）<br>＊初回のみ連絡必須、 子ども活動のみ会員限定
SNS		X auti_tokushima　Facebook Npo法人オーティの会		

活動内容			
	○ 自由交流	講演会	集団療法
	○ 学習・情報収集	SST	○ レクリエーション
	○ 心理教育	ペアトレ	その他

備 考　複数会あり、HP参照。
クローバー（当事者会)、カンガルークラブ(家族会）

高知県　「当事者交流会」高知大人の発達障害当事者の会

当事者会

開催場所		公共の施設		
連絡先		hattatsu.kochi@gmail.com	支援先のHP	なし
参加条件	当事者会	発達障害特性の当事者<br>家族やパートナー（付き添い）	開催	月に1回／対面
	家族会	開催なし	参加費	無料
	診断の有無	なし	参加方法	事前連絡必須（予約・連絡必須）
SNS		X hattatsu_kochi　Instagram hattatsu_kochi		

活動内容			
	○ 自由交流	講演会	集団療法
	学習・情報収集	SST	レクリエーション
	心理教育	ペアトレ	その他

備 考

福岡県　発達障がい当事者研修会よりみち

当事者会

開催場所	市の公共施設		
連絡先	なし	支援先のHP	なし
参加条件 当事者会	発達障害特性、成人の当事者	開催	月に1～2回／対面
参加条件 家族会	開催なし	参加費	有料500円
参加条件 診断の有無	なし	参加方法	直接訪問可（予約・連絡不要）<br>＊初回のみ連絡必須
SNS	X @junneworder		

活動内容	○ 自由交流	講演会	集団療法
	学習・情報収集	SST	レクリエーション
	心理教育	ペアトレ	○ その他（ボードゲーム部、集中部）

備 考　宮崎県にも同団体あり

長崎県　NPO法人 発達障がいお悩み預かり所 かぎしっぽ

家族会　専門家のサポート体制あり

開催場所	主に長崎市		
連絡先	hattatsu.kagishippo@gmail.com	支援先のHP	https://kagishippo.page
参加条件 当事者会	開催なし	開催	月に1回／対面、オンライン
参加条件 家族会	特記すべき条件なし（当事者も可）	参加費	有料500円
参加条件 診断の有無	なし	参加方法	直接訪問可（予約・連絡不要）<br>＊初回の連絡も不要
SNS	X @kagishippo_npo　Facebook NPO法人 発達障がいお悩み預かり所 かぎしっぽ　Instagram hattatsu.kagishippo		

活動内容	○ 自由交流	講演会	集団療法
	○ 学習・情報収集	SST	レクリエーション
	心理教育	ペアトレ	その他

備 考

熊本県 ｜ 発達障がい者・家族の会（熊本）『プリズム』

当事者会　専門家のサポート体制あり

開催場所	熊本市障がい者福祉センター、公民館、老人ホームなど<br>熊本県熊本市西区		
連絡先	080-8554-9244<br>hinokuni.km.@gmail.com	支援先のHP	https://www.prism-kumamoto.com/
参加条件 当事者会	発達障害特性の当事者<br>家族（付き添い）	開催	月に1回／対面
参加条件 家族会	開催なし	参加費	有料　本会員200円<br>準会員500円（1回につき）
参加条件 診断の有無	なし	参加方法	事前連絡必須（予約・連絡必須）
SNS	X @prism_kumamoto　Instagram kumamoto.prism		

活動内容

- ○ 自由交流
- ○ 学習・情報収集
- ○ 心理教育
- ○ 講演会
- SST
- ○ ペアトレ
- 集団療法
- レクリエーション
- ○ その他（代表による講演会、個別に電話相談）

備 考

大分県 ｜ 「みっくす交流会」「親子の会」@はーもにあかふぇ

当事者会、 家族会

開催場所	野口ふれあい交流センター／別府市男女共同参画センター「あす・べっぷ」<br>大分県別府市		
連絡先	050-3692-9388<br>harmoniacafe2019@gmail.com	支援先のHP	https://www.kokuchpro.com/group/harmoniacafe/
参加条件 当事者会	発達障害特性の当事者<br>特記すべき条件なし（付き添い）	開催	交流会：月に1回／対面<br>親子の会：2ヶ月に1回（偶数月）／対面
参加条件 家族会	特記すべき条件なし<br>＊ただし、当事者は発達障害特性	参加費	有料300円
参加条件 診断の有無	なし	参加方法	事前連絡必須（予約・連絡必須）
SNS	X @harmonia_cafe		

活動内容

- ○ 自由交流
- ○ 学習・情報収集
- 心理教育
- ○ 講演会
- SST
- ペアトレ
- ○ 集団療法
- レクリエーション
- その他

備 考

宮崎県 ｜ 成人発達しょうがい当事者会 a tempo

当事者会　専門家のサポート体制あり

開催場所	都城市内あるいは宮崎市内<br>〒885-0035 宮崎県都城市立野町3764－1 南九州大学 園芸福祉研究室		
連絡先	090-1159-8741<br>nhayashi@nankyudai.ac.jp	支援先のHP	なし
参加条件 当事者会	特記すべき条件なし	開催	不定期（開催しない年もある）／対面
参加条件 家族会	開催なし	参加費	無料
参加条件 診断の有無	なし	参加方法	直接訪問可（予約・連絡不要）<br>＊初回の連絡も不要
SNS	なし		

活動内容		
○ 自由交流	○ 講演会	○ 集団療法
○ 学習・情報収集	○ SST	○ レクリエーション
○ 心理教育	○ペアトレ	その他

備 考

宮崎県 ｜ 宮崎県高鍋町発達障がい児・者親の会　キャンパス⭐きっず

家族会　専門家のサポート体制あり

開催場所	〒884-0002 宮崎県児湯郡高鍋町北高鍋956-3（事務所）		
連絡先	090-8831-0364<br>campus-takanabe@gmail.com	支援先のHP	なし
参加条件 当事者会	開催なし	開催	定例会月に1回／ハイブリッド
参加条件 家族会	特記すべき条件なし（当事者も可）	参加費	無料
参加条件 診断の有無	なし	参加方法	直接訪問可（予約・連絡不要）<br>＊初回のみ連絡必須
SNS	なし		

活動内容		
○ 自由交流	講演会	集団療法
○ 学習・情報収集	SST	レクリエーション
○ 心理教育	ペアトレ	○ その他（専門家による勉強会）

備 考　不定期単発で子供達（20代）で企画実施する交流会あり（ex.クリスマス会）

沖縄県 ｜ NPO法人わくわくの会

事業所　専門家のサポート体制あり

開催場所		「さぽーとせんたーｉ」　〒902-0063 沖縄県那覇市三原2-6-1 2F<br>「さぽーとせんたーｉから」　〒903-0805 沖縄県那覇市首里鳥堀町4-106-4		
連絡先		098-987-1167（児童）/ 098-882-4266（成人）<br>wakuwakunokai@gmail.com	支援先のHP	https://wakuwakunokai.com/
参加条件	当事者会	発達障害特性の当事者<br>特記すべき条件なし（付き添い）	開催	ペアトレ年に20～30回、茶話会年に1～2回、大人の当事者会2ヶ月に1回／対面、オンライン<br>＊ペアトレはオンラインの時もあり
	家族会	開催なし	参加費	無料（別途資料代あり）
	診断の有無	なし	参加方法	事前連絡必須（予約・連絡必須）
SNS		Instagram: wakuwakugroup		

活動内容

○ 自由交流	○ 講演会	集団療法
○ 学習・情報収集	○ SST	○ レクリエーション
○ 心理教育	○ ペアトレ	その他

備考

子どもは相談内容による。
「さぽーとせんたーｉ」は主に児童、「さぽーとせんたーｉから」は主に成人が対象。
